# Notes on the Management of Retention of Urine Depending upon Strictures of the Urethra

**Published:** 1872-11

**Authors:** J. W. S. Gouley

**Affiliations:** Surgeon to Bellevue Hospital, N. Y.


### Notes on the Management of Retention of Urine depending upon Strictures of the Urethra.

By J. W. S. Gouley, M. D., Surgeon to Bellevue Hospital, N. Y.

"Wishing to contribute my portion to the already abundant testimony in favor of the conservative treatment of urethral stricture and its complications, and to enter my protest against the frequent resort to cutting and puncturing operations before the simpler and safer methods have been fairly tried, I shall venture to jot down a few suggestions regarding the management of very narrow strictures, impassable to ordinary instruments, and complicated with retention of urine.

If patients suffering from this disease were in the habit of consulting their medical advisers in its early stages, we should seldom be called upon to relieve retention of urine from stricture; but unfortunately they come too late, or the surgeon is summoned to treat the patient under the most unfavorable circumstances—finding either an utterly impassable stricture demanding an immediate cutting operation, often difficult and prolonged, or rupture of the urethra behind the constriction, with extravasation of urine, and perhaps extensive gangrene.

Usually, when the medical attendant fails to reach the bladder with small catheters or bougies in an urgent case of retention, he resorts to one of three operations—puncture of the bladder, external perineal urethrotomy, or incision of the urethra behind the stricture after the plan of Mr. Edward Cock.

The various modes of puncture of the bladder, besides being sometimes extremely dangerous, are not directed to the cure of the stricture, but only to the relief of the distended bladder, leaving the patient in danger of another attack of retention after the removal of the canula. The second and third methods are excellent, provided the stricture be impassable to all manner of slender instruments, or be utterly undilatable, or accompanied by abscess or extravasation of urine. But if there be no complication besides retention, I can conceive of no excuse for exposing the patient to the dangers of any cutting or puncturing operation. One of the most frequent causes of failure of catheterism in very narrow strictures is eccentricity of the orifice of the constriction. If this be borne in mind, many strictures supposed to be impassable will readily be entered by properly-constructed fine bougies. It seems strange that so little heed is given to this, inasmuch as the attention of the profession was called to it by Benjamin Bell, who nearly eighty years ago introduced the abruptly-bent bougie for the penetration of eccentric strictures; and in our own time by Leroy d'Etiolles, who devised the spiral instrument for catheterism of eccentric and of tortuous strictures, and who published many cases successfully treated according to his method.

Though the soft instruments of Leroy answer well in many cases, still I have often seen them fail. On account of their too great flexibility and want of spring they frequently coil in front of the obstruction.

For these reasons I have for many years used in preference slender probe-pointed shafts of whalebone of the length of ordinary bougies, and from half to two thirds of a millimetre in diameter. The points of these delicate instruments retain the shape given them provided they be dipped into boiling oil and suddenly cooled in water. The mode of using them is briefly as follows: after having explored the urethra with a bulbous bougie and ascertained the seat of obstruction, the urethra should be filled with warm lard or olive-oil; then if the stricture be eccentric, a bougie with Bell's bend is introduced, with the point directed toward the floor of the canal, in order to avoid entering the *lacuna magna*. The direction of the point may be changed as soon as it has passed beyond the lacuna; but should it enter another lacuna it should be a little withdrawn, turned aside, and carried along until it encounters the final obstruction. From this moment it becomes an explorer in search of the orifice of the stricture, to find which isnot, as has been asserted, "a mere matter of chance," but a procedure requiring very light fingers, with great delicacy of touch, and much experience and skill. To be sure of entering the mouth of a narrow eccentric stricture the point of the bougie should be kept in contact with the urethral wall, while a slight back-and-forth movement is given the instrument until the stricture is entered or the whole circumference of the canal is explored. The instrument should be bent at both ends in exactly opposite directions, that the distal may indicate the position of the vesical extremity, and consequently the situation of the orifice of the stricture. After the bougie has passed the stricture it should be movable back and forth; otherwise it is almost certain that its point is caught in one of Cowper's ducts, or in one of the many enlarged lacunæ in the ampulla behind the stricture, or in the utriculus, or else in one of the ejaculatory ducts.

At this stage of the peoceeding it is necessary to exercise much caution and gentleness in disengaging the instrument from its faulty position, as any undue force will give rise to a false passage or excite inflammatory action, which may extend to Cowper's gland or to the testicle. These accidents are just as liable to occur from the use of ordinary capillary bougies. By withdrawing the instrument a quarter of an inch, or even less, and giving it a slight rotary movement to change the direction of its point, and then pushing it gently onward, the obstacle will be surmounted and the bladder entered, which is known by the freedom of the point of the bougie on repeating the back-and-forth movement. Having reached the bladder, the bougie serves as a conductor upon which a peculiarly-constructed sound is made to glide and dilate the canal, and also straighten it if the stricture happens to be tortuous.

This last-named instrument was devised by me in 1864, and I have since used it very much and with great success. It is a grooved and conical steel sound, with a canal one eighth of an inch in length at the vesical extremity, and with a curve equal to one fifth of the circumference of a circle three and a quarter inches in diameter. The smallest (No. 3) is one and a half millimetres in diameter at the point. I have had larger ones (to No. 15) made to fully dilate strictures complicated with false routes, and have named them *tunneled sounds*. When a capillary whalebone guide has been passed through a tortuous or an eccentric stricture and has entered the bladder the free end is slipped through the tunnel of the smallest sound, which is carried down to the obstacle—precaution being taken to keep the guide in the groove of the staff—held in firm contact with it, and in a few moments the instrument will be got through, but no foree nor undue pressure should be used. It is desirable, after having accomplished this much, to carry on dilatation rapidly at the same sitting, to four or five higher numbers, to guard against the possibility of retention of urine from too great inflammatory swelling, and then to relieve the distended bladder by means of a tunneled catheter. The stricture should afterward be treated by gradual dilatation, and no other method should be thought of unless dilatation should fail after a thorough trial.

In 1869 I adapted the tunneled principle to the one-eyed English gum catheters, which I have employed with great satisfaction in many instances. The whalebone guide is passed through the vesical extremity of the instrument, which is open, and out of the eye. Their use is especially indicated when, after having made rapid dilatation of a narrow stricture, it is found necessary to retain a catheter for a few hours."—*American practitioner.*

